# Ethical, regulatory, and practical barriers to COVID-19 research: A stakeholder-informed inventory of concerns

**DOI:** 10.1371/journal.pone.0265252

**Published:** 2022-03-24

**Authors:** Bryan A. Sisk, Kari Baldwin, Meredith Parsons, James M. DuBois

**Affiliations:** 1 Division of Hematology/Oncology, Department of Pediatrics, Washington University School of Medicine, St. Louis, Missouri, United State of America; 2 Division of General Medical Sciences, Bioethics Research Center, Department of Medicine, Washington University School of Medicine, St. Louis, Missouri, United States of America; Faculty of Health Sciences - Universidade da Beira Interior, PORTUGAL

## Abstract

**Introduction:**

SARS-CoV-2 (COVID-19) has caused death and economic injury around the globe. The urgent need for COVID-19 research created new ethical, regulatory, and practical challenges. The next public health emergency could be worse than COVID-19. We must learn about these challenges from the experiences of researchers and Research Ethics Committee professionals responsible for these COVID-19 studies to prepare for the next emergency.

**Materials and methods:**

We conducted an online survey to identify the ethical, oversight, and regulatory challenges of conducting COVID-19 research during the early pandemic, and proposed solutions for overcoming these barriers. Using criterion-based, convenience sampling, we invited researchers who proposed or conducted COVID-19 research to complete an anonymous, online survey about their experiences. We administered a separate but related survey to Institutional Review Board (IRB) professionals who reviewed COVID-19 research studies. The surveys included open-ended and demographic items. We performed inductive content analysis on responses to open-ended survey questions.

**Results:**

IRB professionals (n = 143) and researchers (n = 211) described 19 types of barriers to COVID-19 research, related to 5 overarching categories: policy and regulatory, biases and misperceptions, institutional and inter-institutional conflicts, risks of harm, and pressure of the pandemic. Researchers and IRB professionals described 8 categories of adaptations and solutions to these challenges: enacting technological solutions; developing protocol-based solutions; disposition and team management; establishing and communicating appropriate standards; national guidance and leadership; maintaining high standards; prioritizing studies before IRB review; and identifying and incorporating experts.

**Discussion and conclusions:**

This inventory of challenges represents ongoing barriers to studying the current pandemic, and they represent a risk to research during future public health emergencies. Delays in studies of a pandemic during a pandemic threatens the health and safety of the public. We urge the development of a national working group to address these issues before the next public health emergency arises.

## Introduction

The SARS-CoV-2 (COVID-19) pandemic has caused death and economic injury around the globe. On January 9, 2020, the World Health Organization (WHO) announced the existence of a novel coronavirus in Wuhan, China [1]. Two weeks later, the United States (US) confirmed the first case of COVID in the country [2]. On March 3, the US President declared a national emergency and instituted a travel ban for non-US citizens traveling from Europe [1]. Later that month, California became the first of many states to institute stay-at-home orders [3]. By May 28, the number of deaths in the US from COVID-19 surpassed 100,000 [4], and by June, the US passed 2 million cases of COVID-19 [2]. The high prevalence of severe COVID-related illnesses in some communities overwhelmed the capacity of healthcare systems and limited available resources for patients with serious illness (COVID-related or not).

As the medical community began to understand the significance of the emerging COVID pandemic in early 2020, high-quality research on this virus became paramount to developing effective mitigation and treatment strategies. At that time, scientists did not know the mechanism of transmission, mortality rate, long-term consequences of infection, or which personal protective equipment could prevent infection. Furthermore, media and political figures began to publicly doubt scientists and promote unproven treatments. To fill this void, researchers were charged with rapidly finding answers to these questions. On April 22, 2020, the National Institute of Allergy and Infectious Diseases (NIAID) issued a strategic plan for COVID-19 research that included 4 priorities: “Improve fundamental knowledge of SARS-CoV-2 and COVID-19”; “Support the development of diagnostics and assays”; “Characterize and test therapeutics”; “Develop safe and effective vaccines against SARS-CoV-2” [5]. That same month, the National Institutes of Health (NIH) announced a public-private partnership to expedite the development of COVID-19 therapies and vaccines [6].

This urgent need for data created new ethical, regulatory, and practical challenges. For example, this demand for research led to overwhelming amounts of protocols of varying quality. A hospital in China reported receiving 41 applications for COVID-19 research over 35 days, which required a four-fold increase in review conferences and a median time to initial decisions of 2.13 days [7]. At our institution, Washington University’s COVID-19 review group had reviewed 146 new COVID-19 research proposals by April 28^th^. As such, institutions needed to devise strategies for prioritizing and consolidating studies [8, 9] and selecting participants for limited trials [10]. To expedite approvals, some argued for identifying and eliminating unnecessary impediments to approval, while ensuring sufficient protections for participants [11]. Additionally, specific ethical and regulatory questions arose: how to distinguish research vs. public health surveillance [12]; necessity of post-mortem consent [13]; ethics of human challenge studies [14–16]; and how to appropriately share biobanking and repository data [17–20].

Furthermore, the research community has grappled with how to ensure rigor and reproducibility in the context of accelerated research; in the early months of COVID-19 research, journals faced a much higher than normal volume of low-quality research and publications, as well as fraudulent reports [21–26]. Additionally, some scholars worried about an emerging COVID-19 research exceptionalism, where the urgency for data overrode the quality of study design [27].

An intrinsic challenge to conducting urgent human research is balancing the need for appropriate procedures to ensure just, respectful, safe, and rigorous research with the need to eliminate as many barriers as possible to the timely and efficient conduct of research. What are the actual barriers to timely and efficient research during a pandemic? Do they arise primarily at the level of institutions or federal regulations? Which domains of oversight generate serious delays or obstacles—for example, the domains of human subjects protections, data use agreements, recruitment, or intellectual property? Are the primary barriers even ethical or regulatory per se, or primarily logistical (e.g., how to do research while keeping research staff safe or how to ensure oversight services when staff may be ill or working remotely)?.

While there is a growing literature on the ethics of COVID-19 research during the pandemic, few contributions are based on primary data from the experiences of key stakeholders. The next pandemic or public health emergency could be even worse than COVID-19. To prepare for the next emergency, we must learn about the ethical, regulatory, and logistical challenges from the experiences of researchers and Research Ethics Committee (called Institutional Review Board (IRB) in the US) professionals responsible for these COVID-19 studies.

In this study, we distributed open-ended surveys to IRB professionals who reviewed, and researchers who developed studies focused on COVID-19 to create an inventory of challenges and barriers to efficient and effective research. We also asked for examples of adaptations or potential solutions to address these challenges. Because regulatory frameworks, institutional policies, and research funding and sponsorship approaches vary widely across nations, we restricted our focus to research in the US, where our institution is located and where we were familiar with survey distribution strategies to reach our target populations. We launched our survey April 21, 2020, one day prior to the publication of NIAID’s strategic plan for COVID-19 research, and as IRBs were scrambling to work remotely while facing a deluge of new pandemic-focused protocols. This context informed our methodology.

## Methods

We have followed the Standards for Reporting Qualitative Research (SRQR) criteria for reporting our methods and findings [28].

We conducted an online survey in the United States to identify the ethical, oversight, and regulatory challenges of conducting COVID-19 research during the early pandemic, and proposed solutions for overcoming these barriers. In this research project, we used a criterion-based, convenience sampling approach to invite researchers who proposed or conducted COVID-19 research to complete an anonymous, online survey about their experiences. We administered a separate but related survey to IRB professionals who reviewed COVID-19 research studies. This anonymous survey was determined to be exempt by the Washington University Human Subjects Research Protections Office (IRB ID#202004115). Participants were provided with study information in recruitment materials and indicated their agreement to participate by clicking on the survey link. The need to obtain informed consent was waived by the ethics committee.

### Survey design

The surveys were designed to be brief to reduce participant burden. The researcher survey had 12-items, and the IRB professional survey had 9-items. The surveys took approximately 5 to 15 minutes to complete. The surveys included open-ended and demographic items. The online surveys were administered using Qualtrics online survey system, which is HIPAA compliant. Participants were not paid for participating.

Participants who completed the Researcher survey were asked to describe their COVID-19 research, any ethical, regulatory or oversight obstacles or challenges they experienced, to propose solutions and provide their recommendations for overcoming these challenges. We collected demographic data from participants about the type of COVID-19 research study they proposed or conducted, the participant population being studied, whether they worked as a clinician, their role on the research team, and if they sought IRB approval for their research or medical innovation. The IRB professional survey asked participants to describe the challenges they faced when reviewing or overseeing COVID-19 research, new ethical or regulatory challenges they experienced, accommodations made for COVID-19 research studies, and advice for IRBs or researchers involved in COVID-19 research. Demographic items asked about the type of IRB they serve, and to describe their role on the IRB.

### Recruitment of survey participants

We used publicly available contact information to identify COVID-19 researchers and IRB professionals in the United States. The Becker Research Library at Washington University assisted our team, building contact lists for researchers using publicly available contact information from public sources (e.g., ClinicalTrials.gov, Scopus, and Embase Conference Abstracts). Researchers’ contact information, including names and email addresses were added to our recruitment list containing 7,106 researcher contacts. We completed an application process with Public Responsibility in Medicine and Research (PRIM&R), to share our project and recruitment information with their members who are IRB professionals. PRIM&R supported our recruitment efforts by posting our recruitment email and project information on their IRB Forum blog page, on their dedicated COVID-19 webpage, and a brief note about our project with a link to the blog post appeared in their electronic newsletter, PRIM&R This Week.

Data collection occurred during the pandemic, between April 21, 2020 and July 30, 2020. First, we sent the recruitment email to Washington University colleagues as part of a pilot study (n = 12 researchers, and n = 6 IRB professionals). The pilot study did not identify any notable areas of concern and we maintained the same survey and recruitment strategy. Following the pilot study, we sent individuals in the researcher recruitment list an initial recruitment email with an anonymous link to the online survey and three recruitment email reminders. We sent individuals in the IRB professionals recruitment list an initial recruitment email with an anonymous link to the survey and two recruitment reminder emails.

To ensure our recruitment invitation reached as many COVID-19 researchers and IRB professionals as possible, we utilized additional research strategies including posting the recruitment invitation with the link to the survey on social media (e.g., Twitter and LinkedIn), asking colleagues and participants to forward the recruitment invitation to interested individuals, and announcing the project on our Bioethics Research Center website.

We removed eight survey responses from the Researcher survey database because they reported they did not conduct or propose COVID-19 research in the United States, and were ineligible to participate. We added two responses from researchers who mistakenly used the IRB professional version of survey to provide information on their experiences. After removing ineligibles, incomplete responses in Qualtrics, and adding the two surveys from researchers who completed the IRB professional version of the survey, N = 211 for the Researcher survey.

We removed one duplicate response from the IRB professional survey database. We removed eleven ineligible survey responses from respondents who reported they had not reviewed COVID-19 research, or who were not IRB professionals who reviewed COVID-19 research in the United States. After removing partials, ineligibles, one duplicate survey, and moving two responses to the researcher survey database, N = 143 for the IRB professional survey.

We cleaned the open-ended text data and removed the extra spaces in sentences, fixed typos and misspelled words, removed bullet and number formatting, spelled out the abbreviations of some words, and added punctuation and capitalization to ensure responses were in sentence format for data analysis. We had initially proposed to analyze these data using SPSS Modeler’s Text Analysis software. However, after 20 hours of consultation and 11 hours of additional work, we determined that a machine-learning approach would not be time efficient absent a previously established vocabulary and hierarchical ontology. As such, we proceeded with manual coding, as described below.

### Data analysis

Open-ended survey responses were exported from Qualtrics into Dedoose qualitative data analysis software. In consultation with all authors, the Principal Investigator and co-investigator (DuBois, Sisk) led codebook development for both stakeholder groups through iterative consensus coding of 69 transcripts (21 IRBs, 48 researchers). Researcher characteristics relevant to codebook development include: One investigator (DuBois) has served on two IRBs (biomedical and social/behavioral) and trains investigators referred for diverse kinds of compliance failures [29, 30]; another investigator (Sisk) is an oncology clinical investigator and expert on communication in clinical encounters [31–33]. Our coding approach employed inductive content analysis to generate an inventory of key categories of challenges experienced by researchers and IRB professionals while conducting or reviewing research on COVID-19 during the early months of the pandemic, as well as recommended accommodations or solutions [34, 35]. Two authors (Baldwin, Parsons) then coded all transcripts in both stakeholder groups. Coders first consensus-coded 90 (42 IRBs, 48 researchers) transcripts for training purposes, meaning that they each coded the same 90 transcripts and identified any disagreements in coding. These coders then resolved disagreements through discussion with the full authorship team. All transcripts were then split between the two coders, who independently coded their assigned transcripts. To ensure ongoing quality of data coding, coders conducted multiple iterative rounds of coding checks by reviewing each other’s coded transcripts. With these iterative rounds of coding, the authors further refined definitions of themes to ensure clarity in application of codes. This process did not lead to major changes in themes, but rather refinement of codebook definitions and code application. Coders kept detailed records throughout coding and held weekly meetings to resolve questions or discrepancies via consensus.

This study was funded by the National Institute of Aging as a supplement to an R01 implementation trial focused on implementing evidence-based informed consent practices; accordingly, during coding, the team paid special attention to challenges that arose with regard to the informed consent process.

## Results

### Participant characteristics

IRB professionals who participated in this study were predominantly from academic centers (87%). Most respondents were IRB directors (53%) or IRB chairs (22%). Researchers who participated were predominantly physicians (63%) and principal investigators (75%). Researchers were leading various types of COVID-19 research projects, including basic science, diagnostic, treatment, epidemiology, and prevention studies (Table 1).

**Table 1:** Participant characteristics.

Researchers (N = 211)	n (%)
**Profession**	
Physician	133 (63%)
Physician Assistant or Nurse Practitioner	1 (<1%)
Other	10 (5%)
Non-Clinician	67 (32%)
**Role on Research Team**	
Principal Investigator	158 (75%)
Co-Investigator	34 (16%)
Clinical Research Coordinator or Manager	7 (4%)
Other	10 (5%)
**Type of COVID-19 Study**	
Basic Science	9 (4%)
Diagnostic	18 (9%)
Treatment	74 (35%)
Epidemiology	49 (23%)
Prevention	18 (9%)
Other	43 (20%)
Population Under Study*	
Existing Data	40 (19%)
General population	48 (23%)
Healthcare workers	47 (22%)
Patients	124 (59%)
No Human Subjects	15 (7%)
Other**	22 (10%)
**Study Received IRB approval**	
Yes	193 (92%)
Type of IRB Request***	
Research Protocol	169 (88%)
Expanded Access Request	3 (2%)
Other	20 (10%)
**IRB Professionals (N = 143)**	
**Type of IRB**	
Academic Medical Center	85 (60%)
Academic Non-Medical Center	40 (28%)
Independent IRB	2 (1%)
Other	16 (11%)
**Respondents Role in IRB**	
IRB Chair	31 (22%)
IRB Director	76 (53%)
IRB Member (Non-Chair)	7 (5%)
IRB Staff (Non-Director)	18 (12%)
Other	11 (8%)

*Responses were not mutually exclusive, so percentages sum to greater than 100%.

** “Other” included students, university employees, family members of patients, social authorities in a developing country, and teachers, among others.

***Percentage calculated based on denominator of 193 IRB-approved studies. The following data were missing values: research team role n = 2; location of research n = 3, IRB approval n = 1; type of IRB request n = 19.

### Ethical, regulatory, and practical barriers to COVID-19 research

IRB professionals and Researchers described 19 types of barriers to COVID-19 research, with the majority of barriers described by both groups of participants (Table 2). Because our purpose was to generate an inventory of considerations generated by key informants and our sampling method does not permit generalizations about the frequency of barriers or solutions, we do not report counts in any tables. However, every theme was mentioned by at least 3 participants, and most themes were mentioned by more than 15 participants in each cohort.

**Table 2:** Identification of challenges and barriers.

Code	Researchers	IRB Professionals
**Policy/Regulatory**		
Conflicting, Changing, Or Unclear Guidance Or Rules	**X**	**X**
Informed Consent	**X**	**X**
IRB Or Other Regulatory Oversight Body	**X**	**X**
Need for Prioritization of COVID-Related Studies	**X**	**X**
Protocol Deviations	**X**	
Distinguishing Research from Clinical Care		**X**
**Biases and Misperceptions**		
Bias within Medical Community	**X**	
Public Misinformation	**X**	**X**
Political Pressures	**X**	**X**
**Institutional and Inter-Institutional Conflicts**		
Cross-institutional Collaborations	**X**	**X**
Coordination within Institution		**X**
Conflicting Interests	**X**	
**Risks of Harm**		
Privacy or Confidentiality	**X**	**X**
Infectious Risk to Research Team	**X**	**X**
**Pressure of the Pandemic**		
Lack of Knowledge, Information, or Data	**X**	**X**
Time Pressure, Urgency, and Workload	**X**	**X**
Staffing and Logistical Challenges	**X**	**X**
Researcher Opportunism	**X**	**X**
Lack of Institutional Resources		**X**

These barriers related to 5 overarching categories: policy and regulatory, biases and misperceptions, institutional and inter-institutional conflicts, risks of harm, and pressure of the pandemic. We present barriers separately for IRB professionals and researchers in Tables 3 and 4, which include excerpts that illustrate the barriers. However, we discuss findings for both groups together in our results narrative to illustrate connections.

**Table 3:** Barriers to research—Researcher excerpts.

Code	Researchers
**Policy/Regulatory**	
Conflicting, Changing, Or Unclear Guidance/Rules	“I pretty much feel like someone is changing plans on us daily while telling us to submit more information each day.” [R024]
Informed Consent	“The major challenge was the informed consent process. In order to minimize staff exposure, and to minimize use of PPE, attempts were made to forgo traditional in-person, paper consent processes. Use of telemed (i.e. video chat) interactions with patients was attempted; however no secure/approved electronic consent process currently exists within the institution. Verbal consent (with witness) was not considered sufficient.” [R199]
Conflicts with IRB Or Other Regulatory Oversight Body	“My study was suspended for 4 weeks, at the height of the epidemic, because of a dose change that the FDA determined required review for an IND, even though they determined that the study was indeed exempt from requiring an IND, as I had argued clearly 4 weeks earlier. Institutional policies were not a barrier at all, except where limited by the FDA.” [R170]
Prioritization of COVID-Related Studies	“Institutional COVID leadership committees lacking equipoise and censoring research of junior colleagues; unclear separation of "administrative leadership committee" and existing IRB process.” [R070]
Protocol Deviations	“We were not able to complete a monitoring visit that was scheduled during the early phase of stay at home orders” [R021]
Distinguishing Research from Clinical Care	“We appear to have two ethical standards for administering experimental and unproven treatments to acutely ill patients. Under the name of "clinical care", hundreds of thousands of patients with COVID-19 have been given Hydroxychloroquine, Azithromycin, Tocilizumab, and other ’experimental’ therapies with no proven safety or efficacy… Our clinical trial attempting to study a drug being used arbitrarily in current clinical practice had to be approved by an IRB, peer review committee, DSMB, NIH, and FDA, during which process more than 100,000 [died].” [R026]
**Biases and Misperceptions**	
Bias within Medical Community	“I have never seen the political bias translate into clinical research bias like it did after Trump praised Hydroxy.” [R179]
Public Misinformation	“Concern for subject safety due to unethical publication of fraudulent studies and unpublished data from the [hospital]. These articles subjected our subjects to unfounded worry and dramatically limited our ability to get research volunteers.” [R179]
	“The main ethical concern was that enrolling patients in RCTs precluded them generally from getting empiric therapies which they may have preferred as they were touted but nonexperts in the media and on social media (e.g., hydroxychloroquine by President Trump). “[R077]
Political Pressures	“We were also made aware that the executive team from hospital administration was concerned with regards to the public perception of treating COVID-19 patients with [this therapy]. They were concerned that the visibility of the [treatment device] itself would deter patients away from the hospital and further impact revenue.” [R210]
**Institutional and Inter-Institutional Conflicts**	
Cross-institutional Collaborations	“Multiple COVID registries with overlapping information exist, but are not necessarily cooperating.” [R110]
**Unique Risks of Harm**	
Privacy or Confidentiality	“Unclear institutional understanding of the risks and liability related to sharing limited data in an emergency situation. While the research focuses on COVID-19, the dataset to analyze includes demographic and clinical information, as well as geolocation. With such a rich dataset, the risks of re-identification of people who have had COVID-19 and the risks of group harm are increased.” [R144]
Infectious Risk to Research Team	“We had to address issues of safety and resource allocation of research personnel to ensure proper and timely conduct of the research while protecting the employees.” [R088]
**Pressure of the Pandemic**	
Lack of Knowledge, Information, or Data	“We need information about this virus. We have very little. If no likelihood for participant harm, it is important to move approval along as quickly as possible so that fact based data are available” [R037]
Time Pressure, Urgency, and Increased Workload	“Given the time sensitive nature of many of these proposals, the delay and obstacles faced is tremendous.” [R172]
Staffing and Logistical Challenges	“However, not having the regulatory staff has been very difficult. Although they work from home, many things are not done, not signed, and there are no easy electronic options. Data management is doable from home, but interfacing with regulatory bodies in the academic structure is hard, so I do lots of secretarial work.” [R108]
Researcher Opportunism	“Too much unregulated research is being done under the pretext of responding to COVID-19.” [R144]
Lack of Institutional Resources	“Tocilizumab supply was limited and expensive. Rather than letting physicians order it without guidance, we decided to ’control’ use by initiating our own RCT (Tocilizumab vs. placebo). However, as the study ’sponsor’, we are told that we (hospital) need to pay for the drug (or charge patient), rather than charging insurance as usual. We are therefore in the process of stopping our trial, which will likely increase our use of Tocilizumab, but at least avoid potential of hospital paying for the medication (or charging patient).” [R212]

**Table 4:** Barriers to research—IRB professional excerpts.

Code	IRB Professionals
**Policy/Regulatory**	
Conflicting, Changing, Or Unclear Guidance Or Rules	“Do individual IRBs have the capability to adapt to new restrictions, keeping in mind the safety of subjects, and investigators. No guidance was provided from the federal government (i.e., OHRP) in a timely manner.” [IRB111]
Informed Consent	“Navigating the best way to consent such a highly infectious patient in isolation (where a physical consent form can’t be used because of contamination risks) and LARs in isolation, all during a time when people are trying to socially distance was and still is an ongoing conversation.” [IRB102]
Conflicts with IRB or Other Regulatory Oversight Body	“We did not feel a single IRB had documented the necessary components for our site on an expanded access protocol. We had to do extra review on our end to cover consent components relevant to our site. We have had a lot of discussion with investigators about how to appropriately conduct consent processes, but all have been able to come up with situationally appropriate consent processes for their studies that meet the conditions of the regulations.” [IRB098]
Prioritization of COVID-Related Studies	“Chaotic since different researchers in the institution plan similar studies or studies with overlapping components. For emergencies such as this, would suggest institutions have one scientific body to decide on which protocols move forward.” [IRB096]
Protocol Deviations	“The largest policy change was related to protocol deviations. We’ve had to become lax on some of the things that would normally, in trend, be viewed as potential noncompliance. For example, changing protocols visits to become virtual visits. For some research, this has made it where some research procedures are not occurring.” [IRB021]
Distinguishing Research from Clinical Care	“I would remind researchers of the therapeutic misconception, which really took hold of our investigator clinicians. They remain sure these double blinded RCTs and open label treatments such as Convalescent Plasma and Remdesivir are effective treatments despite the lack of evidence. This misconception has led to challenges in the approval and IRB discussion process.” [IRB147]
**Biases and Misperceptions**	
Public Misinformation	“Dealing with national media coverage of investigational drugs (e.g., hydroxychloroquine) which leads to requests for trials at our institution which may or may not be warranted. This was discussed at several meetings (e.g., is a single-site trial at a small institution which is not powered to detect a difference warranted, when a similar multi-site trial may be ongoing which is powered to detect a difference).” [IRB060]
Political Pressures	“In my 20+ year career in research ethics and compliance, this is the most institutional pressure I’ve ever felt to approve research, not just in a timely manner but in the way it came in, even if counter to the regulations. Understanding that these are different times does not mean we can erase the foundations of good research practice. It is a very slippery slope to follow.” [IRB089]
**Institutional and Inter-Institutional Conflicts**	
Cross-institutional Collaborations	“Our institution wanted to participate in the [external study], which had already been reviewed and approved by [external] IRB. There was initial confusion regarding whether a reliance agreement was required (The protocol clearly said it was not), and whether our local IRB needed to convene to review the protocol, which is a requirement of our local IRB policies. The IRB Chair concluded that the urgency of the situation meant that the EAP could proceed so long as the full IRB was informed at their next meeting.” [IRB052]
Coordination within Institution	“Suddenly the IRB became the front not only for the HRPP but for new institutional requirements: Occupational Health and Safety, various data review committees, privacy issues, and departmental approvals to open new studies. As departments were taxed in terms of time and resources, department approval became crucial and the IRB became the gatekeeper. New committees were formed at the institutional level with no clear process or chronology for incorporating such reviews and the IRB dealt with many, many angry researchers and research staff who were frustrated by new and additional requirements.” [IRB142]
**Unique Risks of Harm**	
Privacy or Confidentiality	“Perhaps the issue of privacy and third party consent, associated with Zoom meetings has been compromised; however, we expect researchers to be sensitive in how these meetings are carried out.” [IRB092]
	“IRB members also seemed to be more willing to bend or stretch requirements given the pandemic even given privacy/confidentiality issues with data that would result, such as returning results to subjects or the mandated reporting required” [IRB047]
Infectious Risk to Research Team	“Risk of exposure to COVID-19 in any person to person interaction has weighed strongly in all deliberations by the IRB since the restrictions were proposed.” [IRB118]
**Pressure of the Pandemic**	
Lack of Knowledge, Information, or Data	“One of the biggest issues has been the fluidity of the situation. Early on, the research and university community may not have fully understood the ramifications of the pandemic, and were therefore a bit slow in developing policy and planning. As restrictions were implemented, and further refined, the HRPP staff and the IRB members were in a sense, scrambling to keep up.” [IRB118]
Time Pressure, Urgency, and Increased Workload	“The number of emergency applications has been overwhelming. Along with that, the number of reviews I have had to go through in the past two months has required a commitment of time I have not had, but have done anyway given the emergency nature. I think we have a highly committed and dedicated IRB including staff and members. IRB members and staff should be given due recognition for the extra burden of work they have taken on.” [IRB101]
Staffing and Logistical Challenges	“Also, having a meeting via Webex with roll call voting was totally new to us. We learned by doing, but it was not easy the first couple of times. And taking minutes in that forum is also challenging. We had to create a system for when someone’s audio might cut out or something technical happened that would cause the meeting to pause until everyone was back online.” [IRB113]
	“There has been no increase in staff and a huge increase in number of protocols (approximately 30%). This has resulted in a doubling of our average turnaround time for minimal risk study review.” [IRB127]
Researcher Opportunism	“Some investigators have been "carpetbagging" the NIH for COVID related funding and this has created challenges for unnecessary and unhelpful research. Just because the money is available, doesn’t mean that it will produce quality research.” [IRB101]
Lack of Institutional Resources	“Research Operations Staff has been reduced due to the cost of the pandemic to our network, leading to some delays as work is redistributed among fewer people.” [IRB147]

Although participants identified myriad barriers, 45% of researchers (94/211) and 8% of IRB professionals (16/143) reported that they did not experience any difficulties. The remaining researchers and IRB professionals identified a multitude of barriers described below.

#### Policy and regulatory barriers

*Conflicting*, *changing*, *or unclear guidance or rules*. Some researchers perceived IRBs having unclear rules or guidelines, or contradicting themselves due to rapidly changing guidance. Additionally, researchers noted conflicting guidance or requirements across two or more regulatory or oversight bodies. IRB professionals described similar challenges in adapting to and complying with federal guidance from the Centers for Disease Control and Prevention (CDC), US Food and Drug Administration (FDA), National Institutes of Health (NIH), Office for Human Research Protections (OHRP), or other federal agencies. Both researchers and IRB professionals experienced challenges related to guidance that was rapidly changing, vague, difficult to interpret, or conflicting with other guidance or regulations.

*Informed consent*. Researchers noted difficulties obtaining informed consent due to infectious risk, incapacitation of research participants and need for legally authorized representatives (LARs), and difficulties transitioning to electronic documentation of informed consent. IRB professionals described barriers to informed consent and the need to adapt processes and policies to allow e-consent and permit LARs to provide telephone consent, among other adaptations. Researchers also described patient-related barriers to informed consent that were created by COVID-19. For example, some research participants struggled with using teleconferencing technologies, or had difficulty hearing on the phone during remote informed consent. Other participants could not get their reading glasses because they were isolated and family professionals could not visit. As such, they could not read the informed consent documents.

*Conflicts with IRB or other regulatory oversight body*. Many institutions established COVID-19 research committees that reviewed all research protocols to prioritize access to COVID-19 patients and institutional resources. These sometimes worked completely separately from IRBs; in other cases, approval from the committee was required prior to submitting a COVID-19 protocol to the IRB. Some researchers described frustrating and antagonistic interactions with oversight bodies, including the IRB, COVID-19 research committees, and FDA. IRB professionals described difficulties in interacting with external IRBs and study chairs and achieving consensus on ethical requirements for research.

*Prioritization of COVID-related studies*. Although many researchers acknowledged the necessity of COVID-19 research committees, several researchers described frustrations with the lack of transparency, inability to appeal decisions, and apparent arbitrariness of some decisions. IRB professionals, however, described the overwhelming amount of incoming research studies and the necessity of these COVID-19 research committees.

*Protocol deviations*. Researchers described how the pandemic led to many protocol deviations, for example, because participants missed follow up appointments, missed signatures on consent forms, or received additional drugs not permitted in the study protocol. IRB professionals described the need to permit flexibility around practices that would ordinarily be considered protocol deviations.

*Distinguishing research from clinical care*. Researchers expressed frustrations about needing to pursue several approvals prior to starting a clinical trial, while others were providing the treatment as “clinical care” and publishing their experiences without any approvals. IRB professionals described challenges separating research activities from clinical care or public health activities, which includes off-label and open-label prescribing, public health surveillance, or uncertainty of when to end a placebo-controlled trial in light of new evidence.

#### Biases and misperceptions

*Bias within the medical community*. Researchers described how bias within the medical community seemed to affect which studies were approved at their institutions, which studies were published, and whether hospital staff felt comfortable administering certain experimental agents or off-label treatments. IRB professionals did not specifically describe this issue.

*Public misinformation*. Researchers described how their research was impeded because the lay public held misperceptions about COVID-19 research. They described members of the public being misinformed about certain treatments, therapies, or procedures, and how this misunderstanding affected their interest in participating in a clinical trial. This misinformation originated both within and outside the medical community. IRB professionals described how the media sensationalized certain treatments, and how this news coverage affected their work.

*Political pressures*. Researchers described how political pressures within their organization affected their ability to research certain topics. Due to the urgency and political sensitivity of COVID-19, IRB professionals noted pressures from the media, institutional leaders, and local and national politicians to approve research studies more quickly, and at times to approve studies they perceived to have methodological or ethical problems.

#### Institutional and inter-institutional conflicts

*Cross-institutional collaborations*. Researchers described challenges in developing collaborations between institutions or hospitals. For example, researchers described delays in study development due to multiple IRBs, the need for data use agreements, or concerns about intellectual property. IRB professionals described challenges related to granting permissions for sharing or linking data. This included issues with sharing data with other researchers or institutions, forming repositories or biobanks, accessing data or specimens from other researchers or institutions, or linking biospecimens to patient data.

*Coordination within institution*. IRB professionals described challenges with coordinating research efforts between multiple divisions within the same institution, such as research teams, COVID-19 review committees, the university legal department, and data security teams. Researchers did not specifically identify this challenge.

#### Risk of harm

*Privacy and confidentiality*. Researchers described the tension between preserving privacy and the need for potentially identifiable data to link research data with patients’ clinical outcomes. The need for potentially identifiable data related to biobanks, COVID-19 test results, and phone tracking to monitor travel and spread of virus, among others. Researchers also needed to return certain results to participants, thus necessitating some form of identification. IRB professionals described how new procedures for remote consent and research created new concerns about privacy and security of data. Furthermore, broad sharing of COVID-related patient data also created privacy concerns.

*Infectious risk to research team*. Researchers noted how they were at risk of contracting COVID-19 when working in patient areas, especially when consenting infected individuals. IRB professionals also described concerns about exposing researchers to infection while conducting research. These concerns were exacerbated due to resource limitations early in the pandemic, such as shortages of gloves, masks, and gowns.

#### Pressure of the pandemic

*Lack of knowledge*, *information*, *or data about COVID-19*. Researchers described how limited knowledge about the novel virus created many unknowns, such as how the virus spreads and which personal protective equipment is effective. IRB professionals described challenges in assessing research proposals due to lack of knowledge about COVID-19, including knowledge about the virus’s prognosis, infection rate, and mortality rate.

*Time pressure*, *urgency*, *and increased workload*. Researchers described the time pressure to develop and implement research projects quickly given the risk of the pandemic and the urgent need for data. IRB professionals described the large volume of protocol and modification submissions, and the urgency to expedite approvals.

*Staffing and logistical challenges*. Researchers noted multiple logistical challenges due to the pandemic, such as remote work, needing to use personal phones to call participants, difficulties completing paperwork, and delays in reaching key staff at other institutions who were working remotely. IRB professionals described staffing and logistical challenges, such as IRB professionals who were clinicians being deployed for clinical duty, difficulties convening a large IRB during the pandemic, and technical struggles (e.g., issues with the transition to remote work, lack of portable office equipment, and limited internet access).

*Researcher opportunism*. Researchers described how some researchers were rushing to propose studies, even if they did not have the expertise or capacity to complete the projects. Also, researchers described how this urgency for data led to some low-quality research and subsequent publications. IRB professionals described how some researchers submitted protocols outside of their research expertise and protocols that contained errors or were hastily written. These poor-quality proposals then required extra time or guidance from the IRB professionals.

*Lack of institutional resources*. Researchers described challenges related to insufficient funding for studies, especially related to the high cost of experimental agents used in clinical trials when the studies were unfunded or supported by institutional funds. IRB professionals described how some institutions had insufficient resources to support IRB staffing, shortages of equipment to support remote work, and lack of personal protective equipment to support safe work and research practices. Staffing concerns were exacerbated by hiring freezes, furloughs, and lack of funding.

#### Potential responses, adaptations, and solutions to challenges

Researchers and IRB professionals described 8 categories of adaptations and solutions to the challenges that arise when trying to conduct research on a pandemic during a pandemic. Their proposed responses were frequently general and could be mapped onto more than one barrier or challenge (Table 5). Additionally, 25% (53/211) of researchers provided praise for IRBs during this challenging time. Researchers described how IRBs were prompt, responsive, adaptive, and easy to work with. Several researchers noted how IRBs provided essential resources and guidance that supported their protocol development and submission.

**Table 5:** Proposed adaptations and solutions.

**Enacting Technological Solutions**	
**IRB**	**Researchers**
“Our submission and review system for the IRB was already electronic/online, so that has not been a problem. And it’s not difficult to hold our meetings using Zoom. In this respect, not much has changed.” [IRB043] “A major issue we ran into was how to obtain consent from this population, as a lot of the eligible subjects are no longer able to consent for themselves. We had to run down the use of e-consent, contacting LARs who are currently isolated as they’ve been in contact with an infected patient, the use of verbal consent, etc.” [IRB102] “Our virtual office hours have helped prepare investigators to prepare for their IRB submissions and think about added measures to keep the research participants protected throughout their studies.” [IRB104]	“While studies allow for alternative consent, we worked with IT/Privacy/Security to enable DocuSign for patients and LARs to sign consent. This removed the burden of people having to print, sign, and scan consents from home. “[R092] “We worked with our IRB to adopt ICF processes that maintained the purpose of the procedure but allowed flexibility with infection control practices, and moved forward with an electronic signature consent process that had been planned but not implemented prior to pandemic.” [R064]
**Developing Protocol-Based Solutions**	
**IRB**	**Researchers**
“Researchers should incorporate contingency procedures in their protocols. For instance, what is the plan if a patient on a project goes from mild symptoms, to serious? Are there plans for testing and monitoring research staff? What is the plan if staff get COVID-19?” [IRB118] “Also, be less specific in the timelines and parameters associated with studies. A degree of flexibility will allow for compliant conduct of the study (having a broader visit window, or allowing for consent either in-person or in remote settings).” [IRB017] “Think about the long game when designing consent—think about putting data and specimens in repositories for future research and for sharing, to minimize the risk of infection spread by minimizing additional subject contact.” [IRB003] “We tried to ’automate’ expanded access requests as much as possible (template consent document, template concurrence letter, etc.) but we still used the regulatory process whereby the Chair had to provide concurrence of the use before the use was approved by the IRB.” [IRB132]	“Providing some template/approved language around switching from in person to virtual research or conducting research in person with adequate precautions.” [R111] “Flexibility in "standard" clinical flow patterns (consent, patient tracking, and follow-up) should remain flexible in order to balance both research participant safety as well as the safety of staff. Innovative screening, evaluation, and consent processes using remote solutions should be encouraged; privacy safeguards should be adapted to allow remote processes whenever possible.” [R199] “Work with systems that are willing to undergo central IRB governance and oversight; otherwise your study will be unsuccessful.” [R069] “I think that there should be an easier process for sharing data resulting from this kind of work. I would like to be able to compare my data with other projects who may be doing similar, but slightly different work without being so worried about ensuring participant confidentiality (though I know this is highly important, but it’s unclear sometime when it is allowable and when it is not depending on the data or who you are sharing with).” [R121] “It would be great if (several) examples of approved IRB protocols could be published on the IRB website so that investigators to learn what types of answers are appropriate and likely to be approved. These examples could be updated to address any specific concerns related to COVID-19.” [R073]
**Disposition and Team Management**	
**IRB**	**Researchers**
“We have actually had to strongly encourage people to take time away as it became apparent they were reaching burnout levels. Some of this may be that a vacation day may not seem to be a vacation day when there is not much to do outside the house. Some of it may be dedication. Some of it might be fearfulness for jobs during a time of mass unemployment and furloughs.” [IRB114] “Our entire IRB Office transitioned to remotely working from home. This blurs the line between home and work-life. At the beginning, quite a few IRB staff members felt as though the workday never ended. To resolve this our leadership discussed during staff meetings and one-one-ones, that it is important to maintain a work-life balance and to not feel compelled to work more than normal office hours.” [IRB076] “Be in constant communication with the study teams (especially those conducting prospective research with the infected population). What would typically work in a normal setting, won’t necessarily work anymore. There will be issues no one could possibly predict that only arise when they’re encountered. Protocols are constantly being amended to find workarounds, and the review turnaround has to be extremely quick. At the end of the day, it’s a group effort, we’re all in this together.” [IRB102]	“Always be thinking ahead as to what potential hurdles are since the need to move quickly was vital during this time. I recommend that the Research Office finds ways to stay essential and visible during this time of need. Be crucial members of the team.” [R105] “Receptivity, openness, communication, and active problem-solving (both ways).” [R030]
**Establishing and Communicating Appropriate Standards**	
**IRB**	**Researchers**
“The regulations did not change with the pandemic. We worked to interpret them in a manner favorable to the circumstances, but still applied them across the board. Most of our challenges were process challenges and not really policy or regulation challenges.” [IRB147] “All members of the committee had some additional work to do create, review, and revise new langauge to support researchers at our institution.” [IRB108] “Creating a new document/FAQ for our IRB after attending trainings and reviewing what many others are doing.” [IRB108] “During a crisis or emergency, it’s important for IRBs to be both flexible and consistent. During the initial pandemic response, information changed weekly (sometimes daily) and institution guidance didn’t always follow best practices (whether it be CDC, the state, or the city).” [IRB120] “My advice to IRBs would be to publish guidance and information for researchers to educate and inform them as procedures and expectations are developed or change.” [IRB121]	“The oversight panel would be good to have defined ahead of time for the next crisis with clear definition of how proposals would be evaluated and prioritized. It was unclear to us what permissions were needed, what materials needed to be submitted, the detail required and what the timelines were.” [R072] “The IRBs should go through ALL their standard procedures to eliminate those which make no sense, particularly those that presume face-to-face physical contact. They should also try to separate the important (what is really dangerous) from the trivial (like the exact wording in which one "invites" rather and "asks" someone to participate in research). I also think that they should dethrone ‘consent’ as the ultimate arbiter of what provides genuine human subjects protection.” [R079]
**National Guidance and Leadership**	
**IRB**	**Researchers**
“From a regulatory point of view, the regulations and ICH-GCP all address documentation of consent under the assumption that a piece of paper with wet signatures will be used. Informed consent in many cases has to be obtained over the phone or teleconference. The FDA has been terrific about getting new guidance posted about this. Obtaining signatures has been a challenge.” [IRB090]	“We need to have a centralized repository for protocols (I know there is one, but it’s sparsely populated). Even better, we need to have a single (national) protocol for drug studies that individual sites can use, with the anticipation the data can be gathered into a meta-analysis or other kind of aggregated study later. Trying to join into a study collaboration is too hard, and hard to find the collaborative; limited number of sites allowed; and hospitals may not be ready to join at the same time. But ability to participate is important. Better to have some structure around therapy than just to wait for results from collaboratives.” [R212]
**Maintaining High Standards**	
**IRB**	**Researchers**
“We have not been lowering the standards by which we review and approve studies.” [IRB011] “I think IRBs need to remember the fundamentals of IRB review and the criteria for approval so that the pressures of a pandemic do not lead to sloppy ethical review.” [IRB097] “Don’t accept garbage submissions that don’t provide any benefit to society or people with COVID. Reject these protocols in total.” [IRB101] “Work closely with current advice from agencies, your peers and trust your own instincts. Stay true to your established policies and practices whenever you can. There is a huge temptation to rush and make exceptions but I am even more concerned that pandemic research has the potential to be sloppy and dangerous if we bend the very rules put into place that are meant to protect subjects involved in scientifically and ethically sound research.” [IRB130]	“Don’t abandoned good science in the enthusiasm to perform research.” [R097] “Researchers appear to have made many errors in the rush for COVID19 publications which have ballooned. Does there need to be a code of conduct for researchers working in a pandemic?” [R113] “It is really important to support quality research, and not to cut corners. That’s true for IRBs/clinical investigators (there should be no short cuts on ethical issues) and also the design of the trials themselves. So much of what has been published so far relies on very poor quality data, when we really need evidence-based recommendations.” [R165]
**Prioritizing Studies before IRB Review**	
**IRB**	**Researchers**
“We have a COVID 19 Response Team for Human Subjects Research that meets every morning to discuss new studies and where they are in the IRB review process. Members of this team also consult with investigators when the IRB Office is notified that they are in the planning stages of research. Researchers are encouraged to collaborate with other researchers who may be doing similar research in order to streamline and reduce participant burnout. There is also a task force of the institution that is led by one of the IRB Chairs who happens to be the Chief of Infectious Disease at our institution. The list of new studies is shared with this committee.” [IRB110] “We have implemented a special COVID modification type that allows it to be triaged quicker, and viewed by our most senior staff. We are asking for the risk analysis before we will review, to ensure appropriate triage and if necessary prep for full board.” [IRB013] “We formed an Emergency Committee that would be on call for COVID studies. We already had a Chair Committee formed which comprised of the Chairs, Vice Chairs, and former Chairs of our two Boards. We took this as an opportunity to not only ensure that COVID projects received a speedy review with these members on call, but also utilized this as a training ground to train members of the Board for leadership roles as Chairs and Vice Chairs” [IRB051] “We developed a COVID Advisory Committee to do a rapid pre-review to ensure that studies were not completely overlapping and that potential participants would not end up being over-recruited.” [IRB145]	“For any high-profile problem likely to have high competition of studies/trials for patients, come up with a committee, review process and list of instiutional priorities to help resolve conflicts and limit burdens to patients and the system/staff/hospital, etc.” [R164] “Having a centralized institutional committee focused on COVID-19 research was helpful so that I could be directed to appropriate resources.” [R073] “Develop a COVID-19 Research Steering committee.” [R176] “Oversight bodies should be given guidelines to review projects. An appeal process needs to be created. There should be transparency with any COVID oversight committees. IRB committees should be able to review oversight committees recommendations. Decisions should not be made based on non scientific anecdotal experience.” [R045]
**Identifying and Incorporating Experts**	
**IRB**	**Researchers**
“The IRB: ensure you have availability to consult with Infectious Disease professionals.” [IRB110] “Staff IRB review committee with persons with IRB and ID expertise.” [IRB131] “Find an Infectious Disease doctor/epidemiologist to chair the special COVID review committee.” [IRB079] “Researchers need help with submissions. A good regulatory person to help with submissions is invaluable. Regulatory personnel in the clinical world… are severely needed and often unappreciated, until an IRB protocol expires.” [IRB051]	“It would be difficult to comply with regulatory requirements without very experienced research staff who can devote time to the project.” [R170] “Researchers should speak with experienced mentors (if available) to help navigate politically challenging situations prior to embarking on a research project.” [R130] “We worked with our privacy, legal, and IT security departments to ensure that HIPAA regulations were followed.” [R092] “We have worked with our infection control committee to come up with innovative ways to transport these patients. We will continue the standard cleaning procedures.” [R025]

*Enacting technological solutions*. IRB professionals and researchers described the importance of developing electronic consent processes due to infectious risks. IRB professionals also described the importance of electronic/online IRB systems to avoid reliance on printed documents or wet signatures. Furthermore, some IRBs utilized videoconferencing to offer researchers virtual office hours.

*Developing protocol-based solutions*. IRB professionals and researchers described the importance of providing standard language to expedite drafting of protocols and approvals, such as consent documents, concurrence letters, and methods language around consent processes. One researcher suggested these standard documents should be posted on the IRB website. IRB professionals and researchers also described the utility of minimizing specificity in protocols to allow flexibility and adaptation in the implementation of a protocol. They also described the importance of planning for future data uses in the initial protocol to support data sharing in the future. IRB professionals encouraged researchers to include contingency plans in their protocols to minimize the number of revisions prior to approval. Researchers also recommended that multisite studies utilize a central IRB and centralized resources whenever possible.

*Maintaining high standards*. Despite this need for adaptability and flexibility, IRB professionals and researchers described the need to maintain ethical and scientific integrity. While both groups recommended bureaucratic short cuts, they were adamant that “flexibility” must not be allowed to increase harms to research participants, diminish the quality of the research, or lead to inaccurate or poor quality publications.

*Managing team well-being and communication*. IRB professionals and researchers both encouraged active and open communication between research and IRB teams, as well as effective communication within teams. IRB professionals also described the importance of clarifying workload expectations and monitoring for burnout within the team. One group strongly encouraged IRB professionals to take time off if they demonstrated signs of burnout given the heavy workload.

*Establishing and communicating appropriate standards*. IRB professionals described the need to interpret guidelines and regulations in a manner that is favorable to expediting research, so long as this approach does not harm participants. However, IRB professionals also described how this flexibility must be balanced with consistency so researchers have clear understandings of expectations. Given the rapid changes in guidance during a pandemic, IRB professionals also described the need to publish guidance routinely to educate researchers about updates. Researchers described the need to remove unnecessary steps in the approval process and to provide clear guidance prior to review to improve the quality of the proposal.

*Developing national guidance and leadership*. IRB professionals described the exemplary responsiveness of FDA in providing guidance during the early pandemic. For example, the FDA posted guidance on how to obtain electronic consent. Researchers described the need for stronger federal or national leadership in developing and prioritizing research protocols. For example, one researcher called for a national repository of protocols and more centralized leadership in prioritizing and funding high-priority multisite trials.

*Appropriately screening and prioritizing COVID-19 studies*. IRB professionals and researchers described the need for multiple levels of prioritization early in a pandemic. At some centers, scientific oversight/advisory boards worked with researchers early in the planning stages of studies, and also reviewed full proposal prior to submission to the IRB. One researcher agreed with the importance of scientific review prior to IRB review, but also called for clearer standards for evaluation and an appeals process. Within the IRB, some centers created subgroups of individuals with regulatory and clinical expertise to prioritize and/or review COVID-related protocols. One site developed a new workflow that expedited modification approvals for COVID-19 protocols.

*Identifying and incorporating experts*. IRB professionals and researchers described the importance of engaging clinical, methodological, and regulatory experts into the protocol development and review processes.

## Discussion

Researchers and IRB professionals in this study identified 19 broad kinds of challenges to performing COVID-19 research during this pandemic. These challenges fell into 5 overarching categories: policy/regulatory barriers; biases and misperceptions; institutional and inter-institutional conflicts; risk of harm; and pressure of the pandemic. Furthermore, participants offered a variety of responses to challenges that fell into 8 categories. Many of the recommended solutions or responses were implemented by institutions. However, some of the recommendations will be challenging to implement without further work, and in some cases, new regulations or resources. In this discussion, we reflect briefly on major successes achieved by IRBs and researchers before turning to some of the challenges most urgently requiring attention.

### Shared missions and effective collaboration of IRBs and researchers

IRB participants and researchers generally praised the way that so many IRB personnel rose to the occasion. We learned of offices that expanded hours, gave priority to the review of COVID-19 protocols, added experts in infectious disease and related matters to provide competent reviews, exercised discretion with protocol deviations and modified consent procedures, and in many other ways facilitated research while enforcing regulations meant to protect research participants. They often accomplished these feats without any additional funding or staffing.

Notably, researchers and IRB professionals identified 13 overlapping challenges, and only 6 categories were unique to one group. Given the common perception of adversarial relationships between IRBs and researchers, this amount of agreement was unexpected and encouraging. Overall, researchers and IRB professionals strove for efficient, safe, and effective research to help the medical community and society combat the COVID-19 pandemic.

### Policy and regulatory barriers

Since research on COVID-19 began in early 2020, significant progress has been made to facilitate responsible research. IRBs and researchers have worked together to find solutions for remote informed consent. IRB professionals have debated and disseminated best practices through webinars and blogs, such as Amp&rsand, the blog for Public Responsibility in Medicine and Research (PRIM&R) [36]. Many sites instituted COVID research committees to screen and prioritize COVID studies, even though the prioritization process created tensions and frustrations at times. Furthermore, in April 2020, FDA created guidance on how to balance safety with protocol deviations [37] and OHRP clarified the scope of public health vs clinical activities [38].

However, IRB professionals and researchers agreed on the need for clear guidance—something IRBs did not always feel they could provide in the earliest days of the pandemic. We recommend that IRBs and federal oversight bodies continue to develop clear policies about allowable protocol deviations, standards for review of expanded access program protocols, and acceptability of electronic platforms for documentation of informed consent.

### Biases and misperceptions

Many IRB professionals and researchers described how public and professional misperceptions impeded research on COVID-19. Many in the US public held misperceptions about COVID-19 diagnosis, severity, and treatment that affected the progress of research. Beliefs about the risks and severity of COVID-19 were partly affected by political ideology, as different information ecosystems provided different analyses and sometimes even different facts. The media likely also contributed to public misperceptions about the efficacy of unproven drugs. Over time, this faith in unproven therapies was compounded by a skepticism about vaccines despite rich data demonstrating safety and efficacy. Some clinicians and researchers also believed in the efficacy of unproven treatments, which led some clinicians to withdraw patients from studies if they were enrolled on the placebo arm [39]. Our results support previous findings that political pressures were influencing COVID-19 research agendas. When President Trump touted the efficacy of hydroxychloroquine to treat and prevent serious COVID-19 infections (without sufficient evidence), there developed an urgency to prove the efficacy (or lack thereof) of this drug. An analysis of data from ClinicalTrials.gov in June 2020 found that 237,000 patients were to be enrolled in trials of hydroxychloroquine, which represented 35% of all participants in COVID-19 clinical trials [40]. Eventually, the RECOVERY study in UK found that hydroxychloroquine was ineffective, although some in the media continue to extol the benefits of this medication for COVID-19.

Researchers might feel powerless when they encounter this multitude of biases, misperceptions, and political pressures that drive research agendas. This experience can also frustrate IRB members who are trying to provide sufficient protections for participants. Given the current media environment and information silos within social media, information and misinformation have equal access to enormous platforms. Many of these problems seem intractable, especially for individuals operating within the research system. Any attempts to limit the spread of misinformation will raise concerns of infringing on freedom of speech. Any centralized national oversight to address institutional biases and politics will raise concerns of government overreach or misplaced authority. Also, centralized authority might simply concentrate political pressures and exacerbate the problem.

Political pressures and misinformation are inevitable in any society. Rather than preventing these pressures, perhaps researchers and IRB members should identify ways to leverage these pressures to positive ends. For example, rather than refusing to develop or approve another study of hydroxychloroquine, perhaps researchers should include hydroxychloroquine as one of several treatment arms to encourage public interest and enhance recruitment. Rather than arguing that IRBs need more time to evaluate proposals, perhaps IRBs can utilize the urgency to argue for more staffing or additional incentives to encourage IRB members to expedite processes to the fullest extent possible. Redirection, rather than resistance, could be a useful approach to counteracting misinformation and political pressures.

### Institutional and inter-institutional conflicts

Some COVID-19 research was impeded because of conflicting interests within or across institutions. Within institutions, the decentralized structure of academic institutions created barriers to collaboration and communication between departments. These conflicting interests were also present across institutions, related to intellectual property, access to patients, invitations to join multisite clinical trials, and access to databases and biorepositories.

Given these myriad barriers, it is unclear if the US academic and healthcare systems can facilitate a timely, coordinated response to future public health emergencies. One could argue for more investment of resources to facilitate coordination, but how should these resources should be allocated? NIH leveraged existing Clinical and Translational Science Award (CTSA) hubs in 2016 to develop Trial Innovation Network with the goal of expediting clinical trials by creating new alliances and minimizing roadblocks to research. This investment in CTSAs and research infrastructure likely improved coordination and prioritization of studies during this pandemic [41], but IRB members and researchers still identified major challenges. The US was flooded with small studies and investigators experienced difficulties joining multi-institutional studies. Addressing these barriers will require regulatory reform and stronger incentive structures for collaborations. Perhaps the NIH could develop a panel that prioritizes a small number of experimental therapies and creates standardized research protocols. The NIH could host a central IRB and pay for experimental agents and staff effort. Any site that demonstrates capacity to perform these studies and recruit sufficient patients could join these clinical trials and access government funding. This funding could be tied to a mandate that sites agree to standard data-sharing agreements and utilize a central IRB at the NIH. Perhaps models could also be developed for patent-sharing among collaborating institutions, as we believe that protecting intellectual property impedes the open sharing of data and resources. Such an approach would address some barriers identified by our respondents while adapting to the decentralized nature of the US academic and healthcare systems.

### Risks of harm

When COVID-19 emerged, scientists had very limited knowledge about how to prevent or treat COVID infections. These unknowns created the potential for novel risks of harm to research participants and research staff. Shortages of personal protective equipment further exacerbated this challenge. However, IRBs and researchers across the country seemed to have taken sensible approaches to ensuring safety of all involved, such as permitting teleconferencing rather than in person visits, accepting e-signatures for informed consent, and making determinations about allowable protocol deviations. Furthermore, federal guidance has encouraged this flexibility and adaptability [37, 38]. Protecting research participants is the primary responsibility of all researchers and IRB professionals [42, 43]. As COVID-19 surged, IRB members and researchers seemed to respond quickly and effectively to ensure the safety of participants and staff while supporting the continuation of essential research. This response represents another major successful collaboration between IRBs and researchers.

### Pressure of the pandemic

The urgent need for data in the pandemic created expectations for IRB professionals and researchers that were difficult to sustain. IRBs experienced challenges with sufficient staffing, coordinating remote work, and adapting to significant increases in workload. Researchers similarly felt an urgency to write and submit protocols to answer essential questions about the virus. These pressures also led to researcher opportunism, where some individuals used the opportunity to publish low-quality studies that might have been driven by self-interest. One group performed a Medline search for COVID-related studies in May 2020 (5 months after the first US case of COVID), identifying more than 15,500 articles [24]. Another group estimated that 137 COVID-related papers had been published daily since February 2020 [26]. This vast amount of publications did not necessarily provide clarity or definite answers. These studies often were poorly designed, observational, anecdotal, and written in short format [24].

The potential harms of expediting publication are evidenced by notable retractions from major journals. By late 2020, 33 COVID-related studies had been retracted, withdrawn, or noted with concern because of either data falsification, methodological concerns, inaccurate interpretation of results, authorship disputes, or participant privacy issues [22]. Beyond personal responsibility of researchers and editors, perhaps journals could create an “expedited review process” that involves review by a smaller collection of standing reviewers or editorial board members. To incentivize these expedited reviews, perhaps content experts could receive remuneration for high-quality, expedited reviews. Furthermore, journals might publish articles with reviewer criticisms. This approach could expedite publication while maintaining transparency about study limitations. Furthermore, perhaps the National Library of Medicine (NLM) might consider funding high-quality, ongoing systematic reviews of data during public health emergencies. Such structured, ongoing evaluations could provide up-to-date understanding of collective knowledge while also highlighting major gaps in the literature. Furthermore, this dedicated funding might expedite the completion of these reviews by providing staff support. NLM could also partner with a high-profile journal to expedite review, publication, and dissemination of the current state of evidence at routine intervals.

### Preparing for the next global emergency

The COVID-19 pandemic has served as a test case for whether the US is prepared to handle a major public health emergency. There have certainly been areas of success. For example, pharmaceutical companies and the federal government collaborated to develop remarkably effective vaccines in record time. Our study also showed how IRBs and researchers overcame some of these barriers by collaborating effectively and taking on enormous workloads and responsibilities for the benefit of society. However, we cannot necessarily rely on heroic individual efforts to save us in the future. These efforts were possible because most IRB members were not infected and did not require hospitalization. We did not require emergency IRB review procedures, but it would be reassuring if the OHRP worked with IRBs to establish procedures for delegating review when necessary and for supporting rapid review of greater than minimal risk research.

However, our study and review of literature also identified several areas of unresolved barriers and challenges that might hamper future responses to public health emergencies, ranging from individual behaviors to structural and regulatory issues. These unresolved questions pose an ongoing threat to the health and safety of the US in future public health emergencies. Efforts to address these challenges must account for multiple levels of influence and contributing factors. Implementation science is a field of study that explores the “constellation of processes intended to get an intervention into use within an organization; it is the means by which an intervention is assimilated into an organization. Implementation is the critical gateway between an organizational decision to adopt an intervention and the routine use of that intervention” [44]. Previously, we argued that using the lens of implementation science in ethics is essential to develop actionable, specific ethical norms [45]. In proposing and developing solutions to this inventory of challenges, these solutions should be evaluated using an implementation framework (such as CFIR) [44] and with ongoing stakeholder engagement.

### Study limitations

This study focused on the U.S. context. While some challenges are likely to generalize to other national contexts (e.g., triaging access to COVID-19 patients), many issues will not. Our open-ended survey approach enabled us to get to the field quickly, by April 21, 2020. However, because such a format is more demanding on participants, we kept the survey very brief, perhaps limiting the amount of information captured. Further, our criterion-based sampling gave us a large sample of key informants; however, it does not allow us to state in a generalizable manner the percentage of people who experience a challenge or endorse a response.

## Conclusion

This inventory of challenges represents ongoing barriers to studying the current pandemic, and they represent a risk to research during future public health emergencies. As we have learned during this pandemic, delays in studies of a pandemic during a pandemic threaten the health and safety of the public. We urge the development of a national working group to address these issues before the emergence of the next public health emergency. Key groups might include PRIM&R, OHRP, HHS, National Library of Medicine, and National Academy of Medicine. If we do not take steps to address these issues now, we might fail the test of the next public health emergency.
